# International expert consensus on a structured approach to robotic multiport right hemicolectomy with complete mesocolic excision and intracorporeal anastomosis

**DOI:** 10.1111/codi.70197

**Published:** 2025-08-10

**Authors:** Marcos Gómez Ruiz, Paolo Pietro Bianchi, Roland Croner, Samson Tou, Klaus Matzel, Anthony G. Gallagher, the RORIHEM Study Collaborative, Carmen Cagigas Fernández, George Chang, Håvard Forsmo, Anthony G Gallagher, Dieter Hahnloser, Jim Khan, Klaus E. Matzel, Danilo Miskovic, Wanda Petz, Ellen Van Eetvelde

**Affiliations:** ^1^ Unidad de Cirugía Colorrectal, Servicio de Cirugía General Hospital Universitario Marqués de Valdecilla Santander Spain; ^2^ Instituto de Investigación Biomédica Marqués de Valdecilla (IDIVAL) Santander Spain; ^3^ Faculty of Medicine University of Cantabria Santander Spain; ^4^ Department of Surgery ASST‐Santi Paolo e Carlo Milan Italy; ^5^ Dipartimeno Scienze della Salute Univesity of Milano Milan Italy; ^6^ Department of General‐, Visceral‐, Vascular‐ and Transplant Surgery University Hospital Magdeburg Magdeburg Germany; ^7^ Department of Colorectal Surgery, Royal Derby Hospital University Hospitals of Derby and Burton NHS Foundation Trust Derby UK; ^8^ School of Medicine, Royal Derby Hospital University of Nottingham Derby UK; ^9^ Department of Surgery Diakoneo Clinic Hallerwiese Nuremberg Germany; ^10^ Faculty of Medicine KU Leuven Leuven Belgium; ^11^ School of Medicine Ulster University Derry UK; ^12^ ORSI Academy Melle Belgium

**Keywords:** colorectal resection, colorectal surgery, complete mesocolic excision, intracorporeal anastomosis, metrics, minimally invasive surgery, proficiency‐based progression, training

## Abstract

**Aim:**

To develop and operationally define ‘performance metrics’ that characterize a reference approach to robotic multiport right hemicolectomy with complete mesocolic excision (CME) and intracorporeal anastomosis (ICA) and to obtain evidence supporting face and content validity through a consensus meeting.

**Method:**

Three expert colorectal surgeons with advanced minimally invasive surgical experience, a senior behavioural scientist and a colorectal surgeon with experience in performance metrics development formed the Metrics Group. Published guidelines, clinical evidence, training materials and unedited videos of robotic multiport right hemicolectomy were used to deconstruct the task—robotic right hemicolectomy with CME and ICA—into defined, observable performance units or metrics (i.e. procedure Phases, Steps, Errors and Critical Errors). The performance metrics were then subjected to detailed review by nine expert colorectal surgeons in a modified Delphi process.

**Results:**

Performance metrics for robotic multiport right hemicolectomy with CME and ICA were deconstructed and described as 15 procedure phases with 124 steps, all of them associated with 146 errors and 136 critical errors. After the modified Delphi process, the agreed performance metrics consisted of 15 procedure phases and 125 steps, 150 errors and 139 critical errors. After discussion, agreed modifications to the metrics an international group of expert colorectal surgeons reached 100% consensus on them, thus providing evidence to support the face and content validity of the metrics.

**Conclusion:**

Robotic multiport right hemicolectomy with CME and ICA can be broken down into explicitly defined procedure phases and steps, with errors and critical errors known as performance metrics. We consider the metrics imperative for the development of a safe and structured training in robotic multiport right hemicolectomy with CME and ICA.

**What does this paper add to the literature?**

The present study is the first to describe the development and the performance metrics for training in robotic multiport right hemicolectomy with CME and ICA using a Proficiency‐based Progression (PBP) methodology.

## INTRODUCTION

The optimal treatment for colon cancer involves, in most of the patients, the surgical resection of the bowel. Resection of the colon can be performed in different ways depending on the level of vascular division or lymphadenectomy. Complete mesocolic excision (CME) is a standardized approach that has reported superior oncological outcomes, mainly for patients with stage III disease [1, 2]. There are several challenges to the implementation of CME surgery, mainly the technical challenges associated with the central vascular dissection and lymphadenectomy. A higher risk of superior mesenteric vein injury has been reported in a meta‐analysis [3]. CME can be especially challenging when performed using a minimally invasive approach, but robotic surgery might be of help to safely implement minimally invasive CME and shorten its learning curve [4].

Different anastomotic techniques, such as intracorporeal anastomosis (ICA) and extracorporeal anastomosis (ECA) can be applied after the resection of the bowel. Although still controversial, ICA seems to be related to lower overall complication rates, shorter time to bowel recovery and shorter hospital stay [5]. Again, one of the limitations to a broad acceptance of ICA seems to be related to the technical challenge in a very sensitive part of the procedure, associated with potential life‐threatening complications. Complications associated with anastomosis can have a significant impact on the patient's clinical, functional and oncological outcomes. Furthermore, postoperative complications create a significant burden to healthcare systems.

Recent evidence has shown the relationship between surgeons’ intraoperative performance and patients' outcomes [6, 7]. Structured training can help in the safe implementation of robotic CME with ICA with potential benefits for our patients in terms of improved oncological outcomes and postoperative recovery, decreasing the risk of vascular injuries during CME surgery and expanding the adoption of ICA [8]. Different approaches can be used to scientifically improve intraoperative performance and potentially patients’ outcomes. Proficiency‐based Progression (PBP) simulation training has a very specific methodology consisting of procedure or skill deconstruction into explicitly defined binary metrics that are then validated [9, 10] and used to establish a proficiency benchmark for training courses. Binary metrics are used during training to provide explicit, constructive and formative feedback to the trainee on their performance. The application of this methodology resulted in a superior performance of the trainees. Recent evidence shows that trainees made 60% fewer errors while being trained with PBP methodology compared with quality assured traditional methodologies [11].

The European Society of Coloproctology with the European School of Coloproctology offer education to surgeons, including training in robotic colorectal surgery (ColoRobotica, www.colorobotica.com). As a part of this project, robotic low anterior resection performance metrics were defined, validated and published [12, 13]. Now, robotic multiport right hemicolectomy with CME and ICA performance metrics have been objectively defined. Evidence supporting the face and content validity of the metrics was established through a consensus meeting (i.e. with a Delphi panel) of experienced and expert colorectal surgeons (senior consultant >10 years colorectal practice and experienced in robotic CME and ICA).

## METHOD

Metric development and stress testing (face and content validation) principles for PBP training are described in detail in the literature [14]. This approach was applied when developing the metrics for robotic multiport right hemicolectomy with CME and ICA and is described below.

### Metrics team

The Metrics Group consists of three experienced colorectal surgeons (PPB, RC, MGR) with a special interest in robotic CME and ICA, a senior behavioural scientist and an education‐training expert (AGG) and a colorectal surgeon who has experience in performance metrics/PBP development (ST).

### Robotic multiport right hemicolectomy with CME and ICA metrics development

A detailed task analysis and deconstruction process were used to deconstruct a reference approach to robotic multiport right hemicolectomy with CME and ICA in discrete, non‐overlapping performance units (phases and steps) [14, 15, 16]. Published written guidelines, clinical evidence, video teaching materials and access to 10 anonymized unedited video recordings of right hemicolectomy with CME and ICA, performed by surgeons with different levels of experience supported the metrics development and procedure characterization process, including potential errors and critical errors. The goal was to characterize a ‘reference’ approach to the procedure. A reference procedure is straightforward and uncomplicated to guide trainees in learning the optimum performance of these procedures. The phases and steps are the same for females and males. Characteristics of a straightforward procedure were agreed between the Metrics Group and validated during the Delphi meeting (Table 1).

**TABLE 1:** Patient selection criteria and procedure‐specific criteria for the ‘reference’ procedure.

Patient selection
Right hemicolectomy, non‐extended right resection.
Body mass index—30 kg/m^2^ or less
American Society of Anaesthesiologist (ASA) 3 or less
Cancer disease, non‐T4b tumour/non‐involving other organs.
Procedure
Robotic right hemicolectomy, non‐extended resection
Sub‐ileal approach
Complete mesocolic excision
Intracorporeal anastomosis, side‐to‐side ileocolic mechanical anastomosis

*Note*: A ‘reference’ procedure for training should be a straightforward, uncomplicated procedure.

A one‐and‐a‐half day and preliminary face‐to‐face planning meeting, one face‐to‐face meeting for metrics identification and definition and the metric stress test were conducted. Videoconferences (a total of 36 h) using Zoom (San Jose, California) and email exchanges were used to complement face‐to‐face meetings for further clarification and definition of the metrics.

At the beginning of the metrics development, the Metrics Group agreed on adopting the definitions listed below, which are published in the literature [17, 18, 19]: *Performance metrics*: Units of observable behaviour which together constitute a stepwise description of a reference approach to a procedure. *Procedure Phase*: A group or series of integrally related events or actions that, when combined with other Phases, make up or constitute a complete operative procedure. *Step*: A component task, the series aggregate of which forms the completion of a specific procedure. *Error*: A deviation from optimal performance. *Critical error*: Major deviation from optimal performance, which is likely to cause harm to the patient or compromise the safe completion of the procedure.

The metrics, therefore, consist of procedural Phases involved in a robotic multiport right hemicolectomy. Each Phase comprises specific Steps required for the completion of that part of the procedure. The importance of the metrics approach in defining these Phases and Steps is that these are observable, explicit and unambiguous. The procedure Step either occurred or did not occur and can be scored as such by an external reviewer with high reliability [20, 21, 22]. Similarly, procedure Errors and Critical Errors were defined associated with Steps within different Phases of the procedure. For Errors, behaviours exhibited by the operator may not necessarily lead to a bad outcome or an event with more serious consequences, but their enactment sets the stage or increases the probability of a more serious event to occur or detracts from the efficient and possibly safe execution of the desired procedure. In contrast, a ‘Critical Error’ is a more serious occurrence and represents operative performance that could either jeopardize the outcome of the procedure or lead to significant iatrogenic damage [15, 17, 18].

**FIGURE 1:**
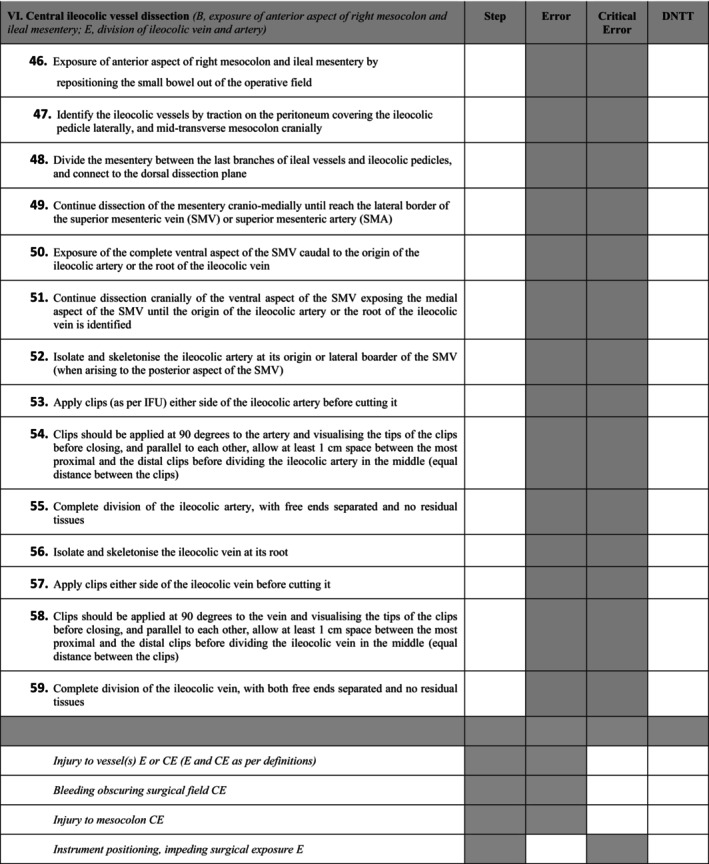
Example of robotic multiport right hemicolectomy with CME and ICA Phase during Steps, Errors and Critical Errors. DNTT, damage to non‐target tissue. Some of the Errors/Critical Errors are shown).

Figure 1 illustrates an example of a procedural Phase (Phase VI. Central ileocolic vessel dissection) characterized for robotic multiport right hemicolectomy with CME and ICA. Once the Metrics Group defined the metrics, they were then used to score four unedited anonymized robotic multiport right colectomy videos performed by different surgeons with various levels of experience. The scorings were performed by the members of the Metrics Group independently. Any difference in the scoring was discussed to identify discrepancies in interpretation or ambiguities in the metric definition. Based on this process, and if agreed upon, changes were made in the metrics, which facilitated the scoring agreement. This process was repeated for each video until the Metrics Group was satisfied with the metrics, and they could be scored with a high degree of reliability (i.e. inter‐rater reliability (IRR) > 0.8) [23, 24].

### Metrics stress testing (face and content validation) with a modified Delphi approach

Once the metrics were defined and characterized, an international panel of expert colorectal surgeons with experience in minimally invasive right colectomy and specifically in robotic multiport right hemicolectomy with CME and ICA was invited to join the Delphi panel to provide objective and independent assessment of the metrics. The panel was chosen for their colorectal surgical experience, experience in robotic right colectomies with CME and ICA and their demonstrated educational interests and commitment.

Thirteen expert colorectal surgeons, including the Metrics Group members from eight countries, and a non‐voting behavioural scientist attended a consensus meeting in St Gallen, 02 December 2024 (Table 2).

**TABLE 2:** Number of surgeons from each country represented in the Delphi panel.

Country	Number of surgeons
Belgium	1
Germany	2
Italy	2
Norway	1
Switzerland	1
Spain	2
UK	3
USA	1
Total	13

A brief overview of the project and meeting objectives was presented. Background information regarding PBP training methodology, prior literature demonstrating the validity of this training approach for procedural specialties, and the specific objectives of the current ‘Delphi panel’ were reviewed [24]. Each Phase of the procedure, the procedural Steps that were included in that Phase, and the potential Errors were presented. It was also explained that the associated metrics had been developed by the Metrics Group for a reference approach to robotic right hemicolectomy with CME and ICA. It was acknowledged that the designated reference procedure might not reflect the exact techniques employed by individual Delphi panellists, but that the operative Phases and Steps presented accurately embodied the essential and key components of the procedure and ‘were not wrong’ [15, 17, 18].

To assess the correlation of the procedure Steps, Errors and Critical Errors before and after the Delphi process, changes were analysed with Pearson Chi‐square (IPM SPSS Statistics for Windows, version 26, IBM Corp., Armonk, N.Y., USA). A *p*‐value of < 0.05 was considered statistically significant.

## RESULTS

The age of the panel ranged from 43 to 66 years; there were three female surgeons included. The combined robotic right hemicolectomies performed or supervised by the Delphi panel were more than 750 cases.

The Metrics Group proposed 15 phases for robotic right hemicolectomy with CME and ICA, each with a defined beginning and end (Table 3).

**TABLE 3:** The beginning and end of the 15 different procedure phases of the reference approach to the robot‐assisted multiport right hemicolectomy with CME and ICA phases, which were presented to the Delphi panel.

Procedure phase	Title	Phase ‐ begins	Phase ‐ ends
I	Patient positioning and preparation	Completion of WHO checklist	Patient is on the table before prepping
II	Preparation of operative field	Creation of a sterile field	Patient is draped
III	Trocar position	Incision/insertion of trocars	Removal of laparoscopic instruments
IV	Docking	Advance the patient cart(s) to the patient	Operating surgeon takes control at the console
V	Mobilization of the right mesocolon and ileal mesentery	Visualize the working end of all three robotic instruments intraabdominally	Complete mobilization of right mesocolon and visualize/exposure of the 2nd/3rd part of duodenum)
VI	Central ileocolic vessel dissection	Exposure of anterior aspect of right mesocolon and ileal mesentery	Division of ileocolic vein and artery
VII	Right colic artery dissection and division when present	Exposure of the anterior aspect of SMV	Division of right colic artery
VIII	Central transection of the right branch of the middle colic artery	Identification of the middle colic artery/trunk	Division of the right branch of the middle colic artery/vein
IX	Dissection of the gastrocolic trunk and transection of the superior right colic vein	Exposure of the lateral aspect of SMV/gastrocolic trunk	Complete division of the superior right colic vein
X	Mobilization of right colon/flexure and division of omentum	Transection of the omentum	Hepatic flexure/right colon is fully mobilized
XI	Division of mesocolon, ileal mesentery and bowel transection	Division of transverse mesocolon	Transection of transverse colon and terminal ileum
XII	Intracorporeal ileocolic anastomosis	Aligning terminal ileum and transverse colon	Completion of anastomosis
XIII	Undocking the system	Robotic Instruments removed	Patient cart(s) removed
XIV	Specimen extraction and wound/ports closure	Make Pfannenstiel incision	Closure of last port site
XV	Transfer patient from operating table to bed	Transfer patient to bed	Patient out of the operating room

During the Delphi meeting, the Delphi panel suggested adding and agreed upon adding a Step in Phase I regarding preoperative imaging scans review, so the trainee has an adequate understanding of the vascular anatomy of the right hemi colon.

During the Delphi meeting, one Step was added, and 24 were modified, resulting in a total of 125 Steps for the 15 Phases of the robotic multiport right hemicolectomy with CME and ICA (Table 4). The added Step was ‘Preoperative imaging scans have been reviewed’ in Phase I. Patient positioning and preparation. Modifications were made in Phases/Steps to make the Steps more explicit and instructive.

**TABLE 4:** Steps before and after the Delphi meeting.

Procedure phase	Title	Steps before Delphi	Steps after Delphi	Added	Deleted	Modified
I	Patient positioning and preparation	8	9	1	0	3
II	Preparation of operative field	5	5	0	0	1
III	Trocar position	17	17	0	0	1
IV	Docking	7	7	0	0	1
V	Mobilization of the right mesocolon and ileal mesentery	7	7	0	0	0
VI	Central ileocolic vessel dissection	14	14	0	0	5
VII	Right colic artery dissection and division when present	5	5	0	0	1
VIII	Central transection of the right branch of the middle colic artery	6	6	0	0	2
IX	Dissection of the gastrocolic trunk and transection of the superior right colic vein	8	8	0	0	1
X	Mobilization of right colon/flexure and division of omentum	6	6	0	0	2
XI	Division of mesocolon, ileal mesentery and bowel transection	13	13	0	0	4
XII	Intracorporeal ileocolic anastomosis	13	13	0	0	2
XIII	Undocking the system	3	3	0	0	0
XIV	Specimen extraction and wound/ports closure	10	10	0	0	1
XV	Transfer patient from operating table to bed	2	2	0	0	0
	Total	124	125	1	0	24

The Metric team identified 146 procedure errors in the 15 phases, and after the Delphi, the total number of procedure errors was 150; 5 errors were added, 1 was deleted and wording was modified in 8 of them (Table 5).

**TABLE 5:** Errors before and after the Delphi meeting.

Procedure phase	Title	Errors before Delphi	Errors after Delphi	Added	Deleted	Modified
I	Patient positioning and preparation	5	4	0	1	1
II	Preparation of operative field	3	3	0	0	1
III	Trocar position	8	8	0	0	1
IV	Docking	5	5	0	0	1
V	Mobilization of the right mesocolon and ileal mesentery	10	10	0	0	0
VI	Central ileocolic vessel dissection	19	20	1	0	1
VII	Right colic artery dissection and division when present	16	16	0	0	0
VIII	Central transection of the right branch of the middle colic artery	20	20	0	0	0
IX	Dissection of the gastrocolic trunk and transection of the superior right colic vein	18	19	1	0	1
X	Mobilization of right colon/flexure and division of omentum	6	7	1	0	1
XI	Division of mesocolon, ileal mesentery and bowel transection	12	13	1	0	1
XII	Intracorporeal ileocolic anastomosis	17	18	1	0	0
XIII	Undocking the system	4	4	0	0	0
XIV	Specimen extraction and wound/ports closure	3	3	0	0	0
XV	Transfer patient from operating table to bed	0	0	0	0	0
	Total	146	150	5	1	8

The procedural Critical Errors were 136 before the Delphi and 139 after the Delphi meeting; 5 Critical Errors were added, 2 were deleted and 7 modified (Table 6).

**TABLE 6:** Critical Errors before and after the Delphi meeting.

Procedure phase	Title	Critical errors before Delphi	Critical errors after Delphi	Added	Deleted	Modified
I	Patient positioning and preparation	8	8	1	0	2
II	Preparation of operative field	4	4	0	0	0
III	Trocar position	9	10	1	0	1
IV	Docking	4	4	0	0	1
V	Mobilization of the right mesocolon and ileal mesentery	12	12	0	0	0
VI	Central ileocolic vessel dissection	14	15	3	2	1
VII	Right colic artery dissection and division when present	12	12	0	0	0
VIII	Central transection of the right branch of the middle colic artery	12	12	0	0	0
IX	Dissection of the gastrocolic trunk and transection of the superior right colic vein	13	13	0	0	0
X	Mobilization of right colon/flexure and division of omentum	7	7	0	0	0
XI	Division of mesocolon, ileal mesentery and bowel transection	15	16	0	0	1
XII	Intracorporeal ileocolic anastomosis	17	17	0	0	0
XIII	Undocking the system	2	2	0	0	0
XIV	Specimen extraction and wound/ports closure	5	5	0	0	1
XV	Transfer patient from operating table to bed	2	2	0	0	0
	Total	136	139	5	2	7

Furthermore, the number of procedure Steps, Errors and Critical Errors before and after the Delphi changes were highly correlated (*Steps*—Pearson correlation coefficient *r* = 0.998, (95% CI, *r* = 0.994–0.999) *p* < 0.001; *Errors*—Pearson correlation coefficient *r* = 0.997, (95% CI, *r* = 0.992–0.999) *p* < 0.001 and *Critical Errors*—Pearson correlation coefficient *r* = 0.997, (95% CI, *r* = 0.991–0.999) *p* < 0.001). On average, there were more Errors after the Delphi with a similar number of Steps and the same number of Critical Errors, although some were modified from Critical Error to Error or added during the Delphi.

After discussion, edits to the metrics agreed and incorporated, the meeting for robotic multiport right hemicolectomy with CME and CIA received 100% consensus from the Delphi panel.

## DISCUSSION

Robotic multiport right colectomy with CME and ICA is a promising procedure for right colon cancer that might have several benefits in terms of postoperative and oncological outcomes in a subgroup of patients. Major concerns regarding CME are the risk of vascular injuries and anastomotic complications, which together with the lack of formal structured training programmes make its implementation difficult. The ESCP with the ESC have created a formal structured training programme in the field of colorectal robotic surgery (ColoRobotica) and recently published the ESCP guidelines on training in robotic colorectal surgery [25, 26, 27].

Evidence shows a strong relation between surgeon's technical skills and patient outcomes [6, 7, 28, 29]. PBP simulation training methodology is a scientific approach that makes technical skill learning a more constructive approach to training with explicit, objective and transparent performance feedback and benchmarking. It trains surgeons on how to do a procedure. More importantly, it also teaches which errors or critical errors are likely to occur in the different Phases/Steps of a procedure and trains surgeons on how to prevent them. PBP training methodology can significantly decrease performance errors during the surgeon skill acquisition phase, down to 60% [11].

The Metrics Group used this methodology to characterize robotic multiport right hemicolectomy with CME and ICA performance metrics (Phases, Steps, Errors and Critical Errors). Three colorectal experts with wide experience in robotic surgery, CME surgery, ICA and trained in PBP methodology, together with a behavioural scientist with extensive experience in surgical training and a colorectal surgeon who has experience with performance metrics/PBP development characterized the procedure.

These performance metrics were analysed and modified by a panel of 13 international expert colorectal surgeons during a modified Delphi meeting, aiming to create metrics that would be useful for trainees. Modifications to metrics were made to make them clear and instructive during training activities. The number of Phases, Steps, Errors and Critical Errors correlated strongly before and after the Delphi meeting, with most of the modifications made in the wording used to characterize the metrics which are used to guide the trainee. A good example of an important modification was made while describing the extension of vascular dissection at the level of the ileocolic artery and vein with 100% agreement by the Delphi panel. This was described in the following way in Phase VI (central ileocolic vessel dissection), Steps 50 ‘Exposure of the complete ventral aspect of the SMV caudal to the origin of the ileocolic artery or the root of the ileocolic vein’, Step 51 ‘Continue dissection cranially of the ventral aspect of the SMV exposing the medial aspect of the SMV until the origin of the ileocolic artery or the root of the ileocolic vein is identified’ and Step 52 as ‘Isolate and skeletonize the ileocolic artery at its origin or lateral boarder of the SMV (when arising to the posterior aspect of the SMV)’.

After discussion by the Delphi panel, voting was obtained on each Phase; reaching unanimous agreement.

During the Delphi meeting, the panel members acknowledged the relevance of having a detailed understanding of the patient's vascular anatomy before starting the surgical procedure [30]. This was added to the performance metrics in Phase I, Step 2 ‘Preoperative imaging scans have been reviewed’.

The PBP method is used not only to create the performance metrics, but also to create performance assessments that can be used to provide constructive feedback in a very explicit and specific way to the trainees. This allows feedback to be more helpful and formative, decreasing the number of errors and critical errors performed by the trainees during their learning curve.

The proposed metrics are for a standard and straightforward procedure, performed in an agreed way by the Metrics Group and the Delphi panel. The aim is to provide a structured stepwise approach to the procedure, although we acknowledge that there is a variety of ways to perform robotic multiport right colectomy, without exposing the SMV, with extracorporeal anastomosis or with a different approach (medial to lateral, cranial first…).

## CONCLUSION

Robotic multiport right hemicolectomy with CME and ICA can be broken down into procedure phases and steps, with errors and critical errors known as performance metrics. These metrics were validated through a Delphi process and will be the backbone of training and assessment of surgeons during the ESCP colorectal robotic surgery training pathway (ColoRobotica), making this training safer for our patients.

## AUTHOR CONTRIBUTIONS

**Marcos Gómez Ruiz:** Conceptualization; investigation; writing – original draft; methodology; formal analysis; funding acquisition; data curation. **Paolo Pietro Bianchi:** Writing – review and editing; investigation; validation. **Roland Croner:** Writing – review and editing; validation; investigation. **Samson Tou:** Writing – review and editing; investigation; conceptualization; methodology. **Klaus Matzel:** Writing – review and editing; project administration; conceptualization; funding acquisition; methodology; supervision; resources; investigation. **Anthony G. Gallagher:** Writing – review and editing; methodology; validation; formal analysis; investigation.

## FUNDING INFORMATION

Intuitive Surgical provided the educational grant for this study to the European Society of Coloproctology and European School of Coloproctology, but did not influence the selection of the experts, the design and conduct of the research, data collection, analysis or the preparation of the manuscript.

## CONFLICT OF INTEREST STATEMENT

PPB, MGR, ST and KM received education grants from Intuitive Foundation and Medtronic. AGG holds education research grants from Medtronic (Dublin, Ireland), the Arthroscopic Association of North America (Chicago, USA) to investigate metric‐based education and training.

## ETHICS STATEMENT

The study protocol was approved by the Institutional Review Board (IRB) at the Region of Cantabria, Spain (CEIm Cantabria).

## Data Availability

The data that support the findings of this study are available from the corresponding author upon reasonable request.

## APPENDIX A Robotic Right Hemicolectomy Metrics Study Group

METRICS—RORIHEM Study Collaborative (Collaborators) are Paolo Pietro Bianchi (ASST‐Santi Paolo e Carlo, University of Milano, Dipatimento Scienze della Salute, Milano, Italy), Carmen Cagigas Fernández (Hospital Universitario Marqués de Valdecilla, Santander, España), George Chang (M.D. Anderson Cancer Center, Houston, USA), Roland Croner (Universitätsklinik Magdeburg A.ö.R, Magdeburg, Germany), Håvard Forsmo (Haukeland universitetssjukehus, Bergen, Norway), Anthony G Gallagher (Faculty of Medicine, KU Leuven, Belgium, School of Medicine, Ulster University, Derry, UK; ORSI Academy, Proefhoevestraat 12, 9090 Melle, Belgium), Marcos Gómez Ruiz (Hospital Universitario Marqués de Valdecilla, Santander, España), Dieter Hahnloser (Lausanne University Hospital, Lausanne, Switzerland), Jim Khan (Portsmouth Hospitals University NHS Trust, UK), Klaus E. Matzel (Department of Surgery, Diakoneo Clinic Hallerwiese, Nuremberg, Germany), Danilo Miskovic (Department of Colorectal Surgery, St Mark's Hospital, Harrow, Middlesex, UK), Wanda Petz (ASST‐Santi Paolo e Carlo, Milano, Italy), Samson Tou (University Hospitals of Derby and Burton NHS Foundation Trust, Derby, UK), Ellen Van Eetvelde (University Hospital Brussels, Brussels, Belgium).
